# Supplementary material for: Are carbon emissions trading and green financial instruments synergistic? -Comprehensive quantitative research based on content analysis
Source: PLoS One. 2024 Mar 7;19(3):e0298601. doi: 10.1371/journal.pone.0298601 (PMC10919601; doi:10.1371/journal.pone.0298601)
Supplement: S1 Data — (ZIP) [file pone.0298601.s001.zip › Data/地级市Z_F工作报告.zip!/─┌├╔╣┼/╢⌡╢√╢α╦╣╩╨/└┤╫╘╣1⁄2╓┌║┼í╛┬φ┐╦╩2╛▌═°í┐_2016.docx]

各位代表：

现在，我代表市人民政府向大会报告工作，请连同《鄂尔多斯市国民经济和社会发展第十三个五年规划纲要（草案）》一并审议，并请市政协委员和其他列席会议的同志提出意见。

一、2015年及“十二五”时期工作回顾

刚刚过去的一年，是我市发展进程中极不平凡的一年，面对复杂形势和严峻挑战，全市上下牢牢把握稳中求进工作总基调，积极适应经济发展新常态，统筹做好改革发展稳定各项工作，较好地完成了市三届人大三次会议确定的目标任务，经济社会发展取得新成效。

（一）经济实现平稳增长。预计全市完成地区生产总值4280亿元，增长8%；公共财政预算收入445.9亿元，增长3.7%；固定资产投资2735亿元，增长14.5%；社会消费品零售总额662亿元，增长8.5%，主要经济指标保持在合理增长区间。出台了培育骨干企业、支持中小微企业、振兴羊绒产业等政策措施。落实电价补贴5.4亿元；新增电力多边交易企业35户，节约用电成本3.3亿元。举办了全国煤炭交易会，签订煤炭供销协议2.56亿吨；协调重点用煤企业增加采购量3180万吨。获批国家和自治区重大项目22项，实施亿元以上项目174项。蒙西至天津南、上海庙至山东特高压电力通道和蒙西至华中铁路等项目开工建设，伊泰精细化学品、中天合创煤炭深加工等项目加快推进，新杭乙二醇、达瑞祥蓝宝石等27个项目建成投产，经济发展后劲持续增强。

（二）转型升级成效明显。设立30亿元转型发展基金，助推产业转型。工业结构持续优化，非煤产业完成投资1535亿元，占工业总投资的82%；实现增加值993亿元，占规模以上工业增加值的48.3%，较上年提高6.1个百分点。农牧业稳定发展，新建现代农业示范基地20万亩，新增市级以上龙头企业32家。文化旅游、金融、物流等产业快速发展，旅游人数、收入分别增长20%和29%，金融存贷款余额分别增长8.3%和4.4%，电商交易额增长10%。第三产业完成投资747亿元，增长22.6%，三产占地区生产总值的比重突破40%，较上年提高0.7个百分点。

（三）统筹发展步伐加快。完成农村牧区“十个全覆盖”工程和环境综合整治投资88亿元，年初确定的3245个项目全部开工建设，改造危旧房3.8万户，硬化街巷道3386公里，改造农村电网8090公里，解决了11.2万人饮水安全问题，300个行政村、2349个自然村实现全覆盖，完成村庄绿化44.7万亩，农村牧区面貌发生巨大变化。品质城市建设扎实推进，完成城市建设投资49.6亿元，入选 “宽带中国”示范城市。交通、水利、电力等基础设施和生态建设力度不断加大，新增铁路运营里程310公里，完成林业生态建设面积151.6万亩，进入国家森林城市行列。

（四）改革创新力度加大。国家资源型经济创新发展综合改革、全国生态文明先行示范区等试点全面推开。在全区率先公布市旗乡三级行政权力清单，率先推行“三证合一”“一照一码”登记制度，新增市场主体3.7万户，增长36.1%。积极推行政府购买公共服务和PPP合作模式。编制了首套自然资源资产负债表。国企改革稳步推进，不动产登记启动实施。深化农村牧区改革，土地草牧场登记确权试点工作基本完成，流转土地草牧场119万亩。实施国家和自治区重大科技项目34项，国家现代农业科技园区获批建设，被评为全国科技进步考核先进市。

（五）民生保障更加有力。完成民生投入468.8亿元，占公共财政预算支出的81%。城乡居民收入达到38131元和14783元，增长9%和10%。累计建成创业园、创业孵化基地26家，城镇新增就业3.2万人。全面落实自治区“三个一”民生工程，零就业家庭实现至少1人稳定就业，949名城乡低保家庭大学生每人每年获得1万元就学补助，为30万低收入农牧户每户发放1吨“暖心煤”补贴。企业退休人员和城乡居民养老金每人每月提高200元和20元，城乡低保标准每人每月提高39元和12元，五保分散供养标准每人每年提高264元。在全区率先实现了跨省市医保实时结算。建设保障性安居工程2.25万套。组建鄂尔多斯应用技术学院，实现我市属地本科高校建设历史性突破。推出了《森吉德玛》等一批文艺作品，有力丰富了群众精神文化生活。

（六）社会大局安全稳定。认真落实自治区银行业支持鄂尔多斯转型发展政策，帮助企业融资689.1亿元，化解银行不良贷款141.5亿元，置换政府存量债务374.8亿元。加大非法集资案件查处力度，妥善处置一批民间借贷案件。通过回购、促销等措施，消化存量商品房294万平方米。大力推进环境整治，取缔关停违法项目94项，整治环境突出问题162个。信访总量和安全生产事故、刑事案件起数同比分别下降7.4%和33.5%、0.8%，社会大局保持安全稳定。

各位代表，2015年全市经济社会保持稳定健康发展，实现“十二五”圆满收官。特别是成功举办第十届全国少数民族传统体育运动会，有力提升了鄂尔多斯对外形象和美誉度，增强了全市各族干部群众的自信心和自豪感，极大地鼓舞了士气、提振了信心、凝聚了力量。

“十二五”时期，是我市经受严峻考验、战胜特殊困难、取得重大成就的五年。全市人民团结奋斗、攻坚克难，推动经济社会发展迈上了新台阶，为全面建成小康社会奠定了坚实基础。

五年来，我们坚持发展第一要务，综合经济实力显著增强。地区生产总值年均增长10.7%，五年累计完成1.93万亿元；公共财政预算收入年均增长13.3%，五年累计完成2038亿元；固定资产投资年均增长15.9%，五年累计完成1.4万亿元；社会消费品零售总额年均增长12.2%，五年累计完成2767亿元。开工建设亿元以上重大项目326项，建成196项。主要经济指标保持全区领先，综合经济实力稳居全国中西部城市前列。

五年来，我们大力推进结构调整，产业转型升级步伐加快。资源型产业向精深加工转化，新增电力装机582万千瓦、煤化工产能901万吨，煤炭就地转化率由11%提高到18%；非煤产业持续做大做强，占规模以上工业增加值的比重提高19.2个百分点。农牧业基础地位更加稳固，粮食产量保持“五连增”，牲畜头数稳定在1200万头只左右。现代服务业蓬勃发展，五年累计完成投资4364亿元。三次产业结构由2.7：58.7：38.6演进为2.4：57.5：40.1。

五年来，我们不断完善基础条件，地区发展环境持续改善。城市功能不断增强，承载能力明显提升，常住人口城镇化率达到73%。蝉联全国文明城市，入选国家园林城市，康巴什新区成为全国首个4A级城市景区。高等级公路通达所有旗区，成为全国铁路通车里程最长的地级市，架设110千伏以上输电线路1万公里，新增供水能力3.5亿立方米。森林覆盖率由23%提高到26.3%，植被覆盖度稳定在70%以上。全面完成“十二五”节能减排任务，万元GDP能耗下降16%，空气质量优良率达到83.4%。

五年来，我们着力保障和改善民生，人民生活水平逐年提高。完成民生投入2080亿元，占财政总支出的80%以上。新增城镇就业17.1万人，登记失业率控制在3%以内。社会保障体系更加健全，部分保障指标处于国内领先水平。新建和回购保障性住房11.2万套。减少贫困人口8.6万人。基础教育水平进入全区前列，医疗服务体系更加完善，成功创建国家公共文化服务体系示范区，成为全国最安全城市、食品安全试点城市、社会管理综合治理优秀市，连续三届获得“全国双拥模范城市”称号。

五年来，我们全面推进依法行政，政府自身建设不断加强。认真开展党的群众路线教育实践活动和“三严三实”专题教育，机关和干部作风明显转变。行政审批事项减少70%，行政事业性收费项目减少53%。积极推进政务公开，旗区全部建立政务服务大厅和公共资源交易中心。严格执行“约法三章”和中央厉行节约规定，“三公”经费持续下降。加大审计监督和行政监察力度，政府系统廉政建设进一步加强。精神文明和民主法治建设深入开展。此外，民族宗教、国防教育、妇女儿童、统计、档案、人防、气象、外事侨务等其它工作均取得新成效新进展。

各位代表，相比五年以前，今天的鄂尔多斯经济实力更加雄厚，基础条件更加完善，干部群众思想更加解放，干事创业劲头更加有力，为创造鄂尔多斯更加美好的明天，汇聚起了更加强劲的正能量。五年的发展历程充满艰辛，五年的发展成果令人振奋。这是自治区党委、政府和市委正确领导的结果，是市人大、市政协大力支持的结果，是全市各族人民合力攻坚、拼搏奋斗的结果，充分彰显了鄂尔多斯人开放包容、诚信友善、不屈不挠、拼搏创新、艰苦奋斗、不断进取的优秀精神品质。在此，我代表市人民政府，向全市各族干部群众，向所有关心、支持、参与鄂尔多斯建设发展的社会各界人士，致以崇高的敬意和衷心的感谢!

我们既要看到成绩，也要看到前进中的困难和挑战。主要表现在：传统产业增长动力不足，新兴产业短期内难以形成有效支撑，稳增长压力持续加大；经济结构不优，产业低端化、产品初级化特征明显，转型任务十分艰巨；改革开放相对滞后，创新引领和人才支撑作用亟待提高；农村牧区基础薄弱，生态环境仍然脆弱，社会事业欠账较多，部分群众生活还很困难；金融、政府性债务、民间借贷、房地产等领域风险还比较突出；政府职能转变还不够到位，不严不实问题还不同程度存在。对这些问题，我们将高度重视，采取有力措施，切实加以解决。

各位代表，五年的实践使我们深刻体会到，必须坚持解放思想、勇于创新，善于把中央、自治区决策部署与鄂尔多斯实际紧密结合，创造性地开展工作，以思想的不断解放推动事业不断发展；必须始终坚持发展第一要务，做大经济总量，转变发展方式，努力实现速度、结构、质量和效益统一；必须突出项目建设，依靠项目推进工作，依靠项目支撑发展；必须坚持改革创新，以改革增活力，以创新拓空间，持续增强发展动力和活力；必须坚持统筹协调发展，形成城乡、区域、经济社会一体化发展格局；必须坚持民生优先，做到发展为了人民、发展依靠人民、发展成果由人民共享。对这些体会，我们将牢牢把握，并在实践中不断丰富和发展。

二、“十三五”时期基本思路和主要任务

“十三五”时期，是全面建成小康社会的决胜阶段。当前，世界经济在深度调整中曲折复苏，国内经济发展进入新常态，能源市场供求关系发生深刻变化，制约发展的不确定不稳定因素仍然较多。但新常态更蕴含新机遇，世界新一轮科技和产业革命正在兴起，国家“一带一路”战略、中国制造2025、“互联网+”行动计划等深入实施，我国产业加快向中高端迈进，自治区加快“五大基地”建设、支持呼包鄂协同发展，这些都为我市提供了难得的发展机遇和市场空间。经过多年的打基础、增后劲、聚潜能，我市经济硬实力、发展软实力、综合竞争力迈上新台阶，支撑经济持续发展的利好因素不断集聚，特别是我们拥有敢于担当、善于作为的干部队伍，拥有奋勇争先、敢闯敢拼的企业家队伍，拥有勤于实践、勇于创新的广大人民群众，这些都为我们推动发展提供了坚强保障。综合分析，今后五年我市仍将处于可以大有作为的重要战略机遇期，完全有基础、有条件、有能力在新的起点上实现更大发展。只要我们始终保持开拓创新的锐气、昂扬向上的士气、敢为人先的勇气，就一定能够开创新常态下鄂尔多斯经济社会发展新局面。

根据市委“十三五”规划建议，今后五年我市经济社会发展的指导思想是：全面贯彻党的十八大、十八届三中、四中、五中全会精神，深入贯彻习近平总书记系列重要讲话和考察内蒙古重要讲话精神，认真落实自治区党委九届十四次全委会议和市委三届七次全委会议精神，坚持“五位一体”总体布局和“四个全面”战略布局，坚持发展第一要务，坚持稳中求进，坚持创新、协调、绿色、开放、共享发展理念，以提高发展质量和效益为中心，着力厚植发展优势，着力破解发展难题，着力推进转型发展，着力补齐发展短板，推动经济社会持续稳定健康发展，把鄂尔多斯建成祖国北疆亮丽风景线上的璀璨明珠。

总体目标是：保持“双高发展”，实现“三个翻番、一个全面建成”。即经济保持中高速增长，地区生产总值年均增长7.5%左右，突破6000亿元，达到6200亿元，较2010年翻一番以上，经济发展继续领跑全区；公共财政预算收入年均增长3%左右，突破500亿元；固定资产投资年均增长10%左右，5年完成2万亿元；社会消费品零售总额年均增长10%左右，突破1000亿元；城乡居民收入年均分别增长8%和9%左右，达到5.6万元和2.3万元，较2010年翻一番以上；发展迈向中高端，产业转型升级取得重大成效，非煤产业占工业增加值比重达到60%以上，三产占地区生产总值比重达到45%以上，经济增长质量效益持续提升，把鄂尔多斯建设成为我国中西部地区极具影响力的区域性中心城市。到2020年，走出一条资源型城市创新创业、转型发展新路子，全面建成较高质量小康社会。

实现上述目标，我们将坚持“五大发展”理念，努力实现“五个突破”。

（一）推进创新发展，在增强发展动力活力上实现更大突破。大力实施创新驱动战略，将创新贯穿到经济社会发展各领域、全过程，深入推进大众创业、万众创新，促进新技术、新产业、新业态蓬勃发展，推动发展方式由要素驱动转向创新驱动，建设国家创新型城市。围绕产业链部署创新链，构建多元发展、多极支撑的现代产业体系，建设国家清洁能源输出主力基地、现代煤化工生产示范基地、铝循环产业基地、西部装备制造基地、面向全国市场的绿色农畜产品生产加工输出基地。到2020年，全市形成3100万千瓦火电装机、800万千瓦新能源电力装机、300亿立方米天然气、300亿立方米煤制气、600万吨煤制油、200万吨氧化铝生产规模；科技研发经费占地区生产总值的比重达到2%以上，高新技术产业占规模以上工业增加值的比重达到20%以上。

（二）推进协调发展，在促进统筹均衡发展上实现更大突破。更加注重产业、城乡、区域、经济社会统筹协调，优化园区、城镇、农牧业、生态空间布局，促进产城融合互动，补齐发展短板，同步推进新型工业化、城镇化、农牧业现代化，着力解决发展中不平衡、不协调、不可持续问题，提升整体发展水平。到2020年，全市户籍人口城镇化水平达到55%左右，金融、文化产业增加值占地区生产总值的比重均提高到5%以上，物流业增加值年均增长12%以上，游客接待量突破2000万人次，加快建设生态、健康、智慧、宜居、宜业、宜游品质城市。

（三）推进绿色发展，在提升生态文明建设水平上实现更大突破。把生态建设作为立市之本，坚持向生态要效益、向绿色要生产力。实施主体功能区战略，全面加强生态系统的规划和保护，有效控制能源资源消耗，大力发展低碳循环经济，促进生态文明建设与经济发展融合互促，建设全国生态文明先行示范区。到2020年，全市森林覆盖率达到28%以上，植被覆盖度达到75%以上，生态环境持续改善，让鄂尔多斯的天更蓝、地更绿、水更净、气更清，筑牢祖国北疆生态安全屏障。

（四）推进开放发展，在拓展合作发展空间上实现更大突破。主动融入国家“一带一路”战略，建设草原丝绸之路经济带重要节点城市。积极参与向北开放桥头堡建设，拓展俄蒙等国际市场，扩大能源化工产品、绒纺制品、装备制造等产品出口规模。加强与京津冀、长江经济带等区域协作，主动融入呼包银榆经济区，促进呼包鄂协同发展。积极承接发达地区高端、环保产业转移，提升对内对外和区域合作水平。到2020年，基本建立起开放型经济新体制。

（五）推进共享发展，在增进民生福祉上实现更大突破。突出普惠性、保基本、均等化、可持续，精心组织实施好重点民生工程。落实就业创业政策，全面掀起鄂尔多斯第三次创业大潮。坚决打赢扶贫攻坚战，实现贫困人口全部稳定脱贫。完善就业、社保、教育、医疗卫生等公共服务体系，社会保险实现法定人员全覆盖，社会事业和保障水平进入全国前列。多渠道增加城乡居民收入，提高公共服务共建共享水平，使改革发展成果更多更公平惠及全市人民，让人民群众有更多获得感、幸福感和自豪感，共同进入全面小康社会。

各位代表，全市人民对幸福生活的向往和期待，就是我们的追求和目标。我们坚信，通过全市各族人民的团结拼搏和不懈努力，“十三五”宏伟蓝图一定实现，一个经济更加繁荣、社会更加和谐、环境更加优美、生活更加幸福的鄂尔多斯必将展现在世人面前！

三、扎实做好2016年各项工作

今年是“十三五”开局之年，也是推进结构性改革攻坚之年，做好今年工作至关重要。我们将深入贯彻中央、自治区和我市经济工作会议精神，落实中央“五大政策支柱”和“五大任务”要求，坚持稳中求进工作总基调，适应经济发展新常态，加快培育发展新动能，着力推进供给侧结构性改革，统筹稳增长、调结构、促改革、惠民生、防风险，持续增强地区发展动力、活力和竞争力，着力保持经济运行在合理区间，实现“十三五”发展良好开局，以优异成绩迎接自治区成立70周年，向全市人民交上一份满意答卷。

全市经济社会发展主要预期目标是：地区生产总值增长7.5%，规模以上工业增加值增长9%，公共财政预算收入增长3%，固定资产投资增长12%，社会消费品零售总额增长9%，城乡居民收入分别增长8%和9%，全面完成自治区下达的节能减排任务。

今年，市政府将重点抓好六个方面工作。

（一）坚持盘活存量与扩大增量并举，大力推进供给侧结构性改革，促进经济稳定健康发展。

调整优化存量经济。积极化解过剩产能。坚持分类指导、分业施策，梳理制定过剩产能清单，制定总体实施方案和分类推进办法，综合运用市场机制、经济手段和法治手段，引导落后产能有序退出。对不符合国家政策要求的产能过剩企业，实行关停并转或剥离重组；对资不抵债、连年亏损、恢复无望的“僵尸企业”，通过资产重组、产权转让、破产清算等措施，依法淘汰退出。努力降低企业成本。开展降本增效专项行动，打好“组合拳”，全力帮助企业去杠杆。通过清理涉企收费、争取税收优惠政策，降低企业税费负担；通过电价补贴、扩大电力多边交易、推进大用户直供电、争取优惠气价等措施，降低企业生产成本；通过协调减免通行费、增加铁路运力等措施，降低企业物流成本；通过展期、续贷、降息等措施，降低企业融资成本；通过清理规范中介服务、培育行业协会等措施，降低企业制度性交易成本。通过“一企一策”，扶持停产半停产企业尽快恢复生产。大力培育中小微企业，新增中小微企业5000户以上。着力稳定煤炭生产销售。抓住国家推广使用低硫环保煤机遇，建立煤炭质量标准体系，完成产地商标认证，打响鄂尔多斯国优煤品牌。落实全国煤炭交易会购销协议，深化与用煤大户和煤炭消费地区合作，开拓新的销售市场。发挥国家级煤炭交易中心、煤博会等平台作用，创新煤炭营销模式。落实国家煤炭行业脱困措施，与周边地区联合限产保价。大力推动煤电、煤化联营，鼓励煤炭企业跨行业兼并重组，增强市场竞争力。加快消化房地产库存。坚持去库存与防风险、化解民间借贷统筹解决。放开城镇落户限制，加速农牧民向城镇转移，鼓励符合条件的外来人口落户，打通供需通道。大力发展城镇服务业和劳动密集型产业，增加人气商气，释放刚性需求。坚持租售并举，多方式、多渠道盘活存量房源。探索“产权共有”模式，消化大户型结构住房。大力推进房地产企业整合重组，引导企业降价促销，增加房地产交易量。全年消化存量商品房300万平方米以上。

培育经济增长新动力。着力抓项目。加强审批对接，争取纳林河矿区、铝循环产业基地、新能源基地等规划，北控煤制气、伊泰200万吨煤制油、中电投烯烃等项目纳入国家“十三五”规划。建立项目推进机制，强化投资和建设力度，全年实施亿元以上重点项目210项，完成投资1600亿元以上，确保26个重点工业项目建成投产。通过向上争取、内部挖潜、市场化运作等手段，全力突破用地、用水、环境容量等制约，保障项目建设需求。突出抓好自治区70周年迎庆项目建设，确保如期建成。着力扩投资。积极释放好的市场预期，引导企业增强投资信心。发挥政府资金引导作用，通过资金整合、引进战略性投资等方式，做大产业发展基金、担保基金规模，争取将市本级产业发展基金扩大到80至100亿元、担保规模扩大到200亿元左右，有效解决产业发展和企业融资难问题。通过企业上市、引进保险资金、推广PPP合作等措施，多渠道、多元化扩大投资，新增社会融资规模700亿元以上。着力抓招商。完善招商引资优惠政策，制定责任清单，开展精准招商、以商招商，通过产业平台招商，引进龙头企业，打造产业集群，营造良好的招商引资生态环境。利用自治区赋予我市的优惠电价政策，加快引进一批新的产业项目，全年引资规模达到580亿元以上。

全力防范和化解风险。强化企业金融风险防控。坚持行政力量与市场手段并举，实施政银企联防，坚决守住不发生区域性金融风险底线。增加信贷投放，缓解资金压力；采取兼并重组、损失核销、资产打包等措施，有效盘活存量资产，降低不良贷款率。严厉打击逃废金融债务行为，保障金融机构合法权益。强化政府性债务风险防控。坚持开源节流与化债相结合，统筹做好财政增收、节支、化债工作。建立现代财源体系，通过培植新税源、挖掘增长潜力、向上争取等措施，提升财政保障能力。通过争取债券置换资金、财政预算安排、资源资本化等方式，统筹做好政府性债务化解工作。强化民间借贷风险防控。严厉打击非法融资和集资诈骗，设立金融、房地产仲裁院，加快案件审理和资产处置进程，鼓励通过实物置换等合理有效方式，化解债务链条，严厉打击“老赖”行为，保障群众合法权益，力争把群众因民间借贷造成的损失降到最低。

（二）坚持调结构与补短板并重，持续推进产业转型升级，提高经济增长质量和效益。

推动工业经济转型。严格控制初级产品产能扩张，突出产品技术创新，大力延伸产业链条，促进产业向中高端迈进。集中打造绿色煤电基地。加快蒙西至天津南、上海庙至山东电力通道建设，力争1600万千瓦配套电源点项目上半年全部开工，开展鄂尔多斯至江苏、蒙西至湘南电力通道前期工作，确保华能巍家峁等电厂建成投产，新增火电装机120万千瓦以上。做好规划对接，争取将我市列为国家煤电铝一体化循环产业示范基地。做大煤基化工产业规模。推动神华煤制油第二三条生产线、中煤大化肥二期、汇能煤制气等项目建设，争取北控等煤制气、伊泰200万吨煤制油、中电投烯烃、渤海化工综合基地等项目尽快核准、开工建设，确保伊泰精细化学品、中天合创煤制甲醇等项目建成投产，新增煤制烯烃产能200万吨、精细化学品120万吨，甲醇就地转化率达到30%左右。大力发展新能源产业。抓住国家支持电力通道打捆外送新能源政策机遇，建设大型风电、光电、光热发电站，打造国家级新能源产业示范基地。稳步扩大常规天然气产量，推进鄂尔多斯至沧州、陕京四线、蒙西煤制气等输气管道建设，提高清洁能源外送能力。促进装备制造业高端配套发展。推进奇瑞、华泰等企业改造生产线，着力引进零部件配套企业，提高本地配套化率，引进配套企业15家以上。利用低成本电价优势，大力发展新能源汽车产业，争取获批生产资质。加快京东方液晶显示器二期等项目建设，做大煤机、化机、风机等设备制造规模，引入企业打造多晶硅全产业链。培育云计算产业集聚优势。制定云计算产业发展路线图，集中打造国内极具竞争优势的云计算产业基地。今年新改建标准化数据机房10万平方米，争取亚马逊、曙光等企业落户，鄂尔多斯至北京专用光缆上半年建成投用。积极改造提升传统产业。落实羊绒产业振兴政策，加快鄂绒工业园区二期、羊绒交易中心等项目建设，巩固国际羊绒产业领头羊地位。推进PVC、陶瓷、冶金、建材等产业技术升级改造，拓展销售市场，让传统产业焕发出新的生机和活力。

大力发展现代服务业。实施现代服务业发展三年行动计划，出台配套扶持政策，推动服务业发展提速、比重提高、水平提升，三产占地区生产总值比重再提高1个百分点。壮大文化旅游业。深入挖掘民族文化资源，大力发展文化创意、影视等产业，推进蒙古源流文化产业园、西部艺术品交易中心建设，打造西部影视产业基地。以建设国家旅游业改革创新先行区为龙头，系统谋划、系列打造旅游业，建设全国休闲度假旅游目的地，打造内蒙旅游、北方旅游、草原旅游龙头。加强景区景点建设，提升完善配套服务能力，建设立体旅游交通网络，推出一批精品经济旅游线路。大力发展智慧旅游，建立旅游公共服务平台、品牌营销中心，与港中旅、携程等大企业合作，强化旅游宣传促销。全年接待游客突破1000万人次。构建现代物流体系。整合物流资源，依托立体交通网络和空港等园区平台，强化与天津物产、中航物流等大企业合作，建设中西部地区有影响力的大宗商品交易中心和区域性物流分销集散地。引进2至3家大型物流企业，争取空港综合保税区和内陆港获批。做大做强金融业。抓好金融综合改革，大力引进银行、保险、证券、金融租赁等企业入驻，加快设立民营银行，完善地区金融体系。实施“互联网+金融+产业” 发展计划，提升金融业支持实体经济能力。抓住股票注册制度改革机遇，大力推进企业上市，构建多层次资本市场。培育健康养老产业。引进优质资源，培育面向全国的健康养老、疗养度假等产业项目，创造低成本、高质量的居家养老环境，吸引旅居养老、生态养老、候鸟式养老人群，建设国家级社会化养老示范基地。发展电子商务新业态。加强与淘宝、京东、苏宁易购等平台对接合作，启动建设2至3家旗区级地方特色馆。鼓励发展城市快递和农村电商产业，引导中小物流企业承接同城配送业务，畅通农畜产品销售渠道，解决农牧民“卖难”问题，推进国家农村电子商务示范县、全区电子商务产业集聚区建设。

提升农牧业现代化发展水平。加快沿黄河、无定河流域现代农牧业综合示范区建设，提升农牧业规模化、特色化、品牌化、市场化发展水平。建立完善土地流转平台，推动土地草牧场向专业大户、农牧民专业合作社、产业化龙头企业等新型经营主体集中，流转土地10万亩、草牧场165万亩以上，新建公司化养殖园区30处、生态型家庭牧场300户、养殖大户300户。大力培育肉羊、肉牛、生猪、家禽等优势特色产业，扩大青贮玉米、瓜果蔬菜、优质牧草种植比例，建设100个产业结构调整示范村和300个产业发展重点村。抓好国家现代农业科技园区建设，实施“藏粮于地、藏粮于技”战略，发展节水型农业，改造中低产田，提高农业综合机械化水平，建设现代农牧业节水基地20万亩。大力发展精品农业，推进绿色、有机、无公害农畜产品认证，培育10个以上国内知名品牌。加大政策扶持力度，培育壮大龙头企业和农牧民专业合作组织规模，新培育15家产业化龙头企业，力争农牧民专业合作组织覆盖50%以上农牧户。

（三）大力改善农村牧区基础条件，推进以中心城区为核心的城镇化建设，提高城乡统筹发展水平。

全力推进“十个全覆盖”工程建设。加强组织领导，强化政策配套，集中力量、集中攻坚、集中突破，完成投资110亿元，改造危房3.79万户，硬化街巷道6674公里，改造农村电网4050公里，解决16万人安全饮水问题，6月底前完成“十个全覆盖”基本工程量，到年底高质量全面完成建设任务，实现功能全覆盖、村庄全覆盖、人口全覆盖。把“十个全覆盖”工程建设与产业发展、结构调整、农牧民增收、基层组织建设、乡风文明大行动结合起来，拓展工程建设内涵，促进农村牧区产业发展可持续、农牧民增收可持续。强化村容村貌整治，完善村庄环卫设施，推进村庄绿化美化，建设美丽乡村。大力推广使用清洁能源，在有条件的地区实施天然气、宽带入户工程，努力让农牧民享受到与城市居民一样的品质生活。通过政府购买服务、群众参与等方式，建立村庄长效管理机制，提升治理水平。

加速推进城市化进程。落实中心城区一体化发展政策措施，率先推行“多规合一”，加速推进基础设施、公共服务、城市管理、社会保障一体化。力争年内实现康巴什设区。加大城市绿化力度，巩固园林城市创建成果，新增绿化面积500万平方米。积极支持帮助企业推动在建楼宇建设，使其尽快达到居住使用条件，形成有价资产进入市场交易，有效解决“烂尾楼”问题。深化城市管理执法体制改革，推进城市建设和管理领域立法。扎实抓好国家智慧城市、信息消费试点和“宽带中国”示范城市建设。开展住宅小区文明创建、提升城市品质攻坚行动，强化物业管理服务，集中整治老旧小区脏乱差问题，努力让城市居民生活得更方便、更舒心、更美好。加快县域城镇化进程，完成中小城镇发展规划编制工作，实施苏木乡镇政府所在地改造提升工程，增强承载和辐射带动能力。

（四）扎实推进改革攻坚，大力促进创新创业，进一步激发地区发展动力和活力。

持续深化改革。认真落实中央、自治区全面深化改革的各项政策措施，加快资源型城市创新发展和全国生态文明先行示范区建设。全面推行“三单”管理，6月底前公布市级部门责任清单，建立公共服务、行政审批单一窗口受理和网上审批平台，力争把我市建成我国西部地区审批最少、服务最优城市之一。强化“红黑名单”管理，建立社会信用信息平台，加大失信惩戒力度。建立城乡统一的户口登记制度。加大煤炭资源配置清理力度，大力推进资源资本化。统筹推进国资国企、财税金融、投融资领域改革，组建资产管理公司，扩大PPP合作规模和政府购买公共服务覆盖面。推进水权、碳排放权和排污权交易，实行居民用水用气阶梯价格制度。加强生态文明制度建设，编制自然资源资产负债表。建立草原土地卫星动态监测平台，做好农村牧区土地和草牧场确权登记颁证工作。

强化创新驱动。建立科技创新服务和技术转移平台，编制重点产业发展技术路线图，着力推动清洁能源、煤化工、先进制造、新材料等产业领域技术攻关。组建现代煤化工产业技术联盟，争取建立国家级煤炭检测中心。实施科技专家进园区进企业和高新技术企业培育计划，9月底前实现国家级高新技术园区挂牌。推进自治区人才改革试验区建设，完善落实人才政策，大力培养产业工人，引进培养实用型人才1.8万人。

推动全社会创业。强化创业载体建设，新建20家创业园、创业孵化基地和10家以上自治区级众创空间试点，在每个旗区打造1个“双创”示范基地。设立创业基金，支持草根创业、微创新、微创业。大力开展对创业团队和创业人群的针对性培训，实施“小老板”培育、中小企业团队打造工程，使大众创业、万众创新蔚然成风。

（五）强化生态建设和环境保护，推进重大基础设施建设，持续改善地区发展环境和条件。

加大生态保护建设力度。坚定不移执行禁牧、休牧、轮牧和草畜平衡政策，严查开荒、偷牧等行为，巩固生态建设成果。实施新一轮草原生态奖补政策，推进京津风沙源治理、天然林保护二期、退耕还林还草等重点工程建设，完成林业生态建设120万亩，治理水土流失180万亩。建设一批绿化苗木、花卉苗圃基地，降低绿化成本，提高绿化效益。

坚决整治环境污染。像保护眼睛一样保护生态环境。严格执行新《环保法》，全面落实大气污染、水污染防治计划，推进棋盘井蒙西等重点区域环境整治，严肃查处环境违法行为。严把项目引进关，坚决拒绝“三高”项目落地。加强城镇和园区污水、垃圾等处理设施建设，实现重点企业环保在线监测全覆盖。建立重污染天气联合监测预警机制，推进饮用水源地保护和中小流域综合整治，切实让群众呼吸上更新鲜的空气、喝上更干净的水。

加强基础设施建设。推进东胜至包头高速改扩建、鄂前旗至银川高速等公路项目建设，新开工高等级公路306公里，建设农村牧区公路3004公里。加快东乌与包西联络线等铁路项目建设，新增运营里程130公里；实施既有铁路客运改造，尽快实现主要城镇铁路客运通达；着力突破高铁“瓶颈”，争取国家启动实施呼准鄂客货铁路改客运专线工程，将包鄂西客运专线列入国家铁路中长期建设规划和“十三五”规划；5月1日前开通呼市至包头至鄂尔多斯动车，填补我市动车空白。实现鄂尔多斯机场国际航空口岸对外开放，开辟国际航线，增开国内航线，建成2至3个通用机场。推进黄河内蒙古段二期防洪工程建设，完善城区和园区供水体系，完成水权转换二期工程，强化非常规水源回收利用，增强水资源保障能力。

（六）更加关注民生，增进人民群众福祉，让老百姓拥有更多获得感。

实施好重点民生工程。全力推进扶贫开发。按照“六个精准”“五个一批”要求，着力解决“扶持谁、谁来扶、怎么扶、如何退”的问题，突出抓好易地扶贫搬迁，坚决打赢脱贫攻坚战。年内确保国家标准线下1.27万贫困人口全部脱贫和杭锦旗区贫旗摘帽。加大对市低保线下贫困人口的帮扶力度，完成建档立卡工作，确保人均收入较上年提高15%以上。努力促进更多就业。提高就业组织化程度，多渠道、多方式开发就业岗位，千方百计保持就业形势稳定。强化就业指导培训，对贫困家庭、下岗职工、残疾人等群体进行免费培训，实现零就业家庭动态清零。落实好援企稳岗补贴政策，引导企业不裁员、不减薪。全年发放小额担保贷款2.5亿元，新增城镇就业3万人，城镇登记失业率控制在3.1%以内。提升社会保障水平。稳步提高各项社会保险待遇，实现自治区范围医疗保险异地结算。继续实施贫困大学生教育救助和低收入农牧户“暖心煤”政策。以回购为主新增保障性安居工程15005套，将符合条件的非本市户籍人口纳入保障范围。更加关注城乡低保户、残疾人等特殊困难群众生活，织密织牢社会保障安全网。

提高社会事业发展水平。优先发展教育事业。统筹配置区域、城乡教育资源，促进教育公平。积极推进“立德强能”、名师培养、学校标准化和安全改造工程，减轻学生课业负担，强化师资培训，新建续建改扩建中小学校和幼儿园67所，确保市三中新校区等建成投用。加快发展高等教育、职业教育、民族教育，整合资源打造一批高等教育重点学科。加强教育信息化建设，提升教育教学整体水平。加快发展卫生事业。深化公立医院改革，破除以药养医体制。加快市中医院住院楼、市妇女儿童医院等工程建设。推进医疗资源整合，提高利用水平。实施骨干医生、全科医生培训和高端医疗人才引进计划，打造一批名科名院。大力发展中蒙医药事业，培育一批名方名药。落实一对夫妇可生育两个孩子政策。大力发展文化事业。实施“十个一”文化工程，巩固国家公共文化服务体系示范区创建成果。强化重点文物、非物质文化遗产、地质遗址保护，实施长城遗址保护工程，完成第一次全国可移动文物普查验收。积极发展体育事业。开展全民健身运动，提高全民健身水平。深化足球事业改革，大力推动校园足球发展。举办好全国青年田径锦标赛等20余项区内外体育赛事活动。

维护社会安全稳定。加大矛盾纠纷排查化解力度，推行网上信访、涉法涉诉信访事项会商转办、疑难复杂信访事项储备金制度，解决一批信访积案。深化平安鄂尔多斯建设，完善立体化治安防控和合成作战机制，建成地方“警务云”体系。严厉打击刑事暴力、电信诈骗等违法犯罪活动，加大禁毒和反恐工作力度，保持社会治安形势稳定。做好社区矫正、法律援助、“七五”普法等工作，提高社会法治意识。严格落实安全生产责任，建设隐患排查治理信息系统，落实专家巡查和分级分类监管制度，强化培训教育，抓好安全监管，防止重特大事故发生。加强应急队伍建设和救援演练，提高应对处置突发事件的能力。积极创建国家食品药品安全城市，确保人民群众“舌尖上的安全”。加强国防宣传教育，推动军民融合深度发展，巩固双拥模范城市创建成果。认真落实党的民族宗教政策，切实维护各族人民团结奋斗、安居乐业的大好局面。

四、加强政府自身建设

实现全面建成小康社会目标，必须加强政府自身建设，不断提高新常态下推动发展的能力和水平。

我们要推进依法行政，做到重法治、严规范。自觉接受人大法律监督、工作监督和政协民主监督，定期向市人大常委会报告工作，向市政协通报情况，认真办理人大代表议案、建议、意见和政协委员提案。加强与各民主党派、工商联、无党派人士和人民团体联系，主动接受社会监督、舆论监督。强化政府立法，在涉及群众切身利益等领域率先出台一批政府规章。加强法律宣传教育，增强公务人员依法行政水平。建立完善行政处罚裁量权基准制度，为权力行使定边界、划红线。推进文明执法，规范执法行为。

我们要加快转变职能，做到强管理、提效能。大力推行电子政务、代办服务和“一站式”服务，让老百姓少跑路、多办事、办成事。加强绩效管理，健全激励考评机制，激发干事创业热情。强化执行力建设，加强督促检查，确保每项工作善始善终、善做善成。严格行政问责，坚决查处慢作为、乱作为、不作为和“庸、懒、散”等行为。坚持问题导向，推行“一线工作法”，到基层一线发现问题、研究问题、解决问题。深化政务公开，及时回应社会关切，提高政府工作透明度和公信力。

我们要持续改进作风，做到勇担当、有作为。践行“三严三实”，驰而不息纠正“四风”。加强调查研究，始终把老百姓冷暖放在心头，真正为群众办实事、办好事。推进工作理念和方式方法创新，着力破解制约地区经济社会发展的深层次矛盾和R题，勇于担当、敢于作为，一心一意谋发展，脚踏实地抓落实，创造性地开展工作，以忠诚、干净、担当的形象取信于民。建设“诚信政府”，对作出的决策一抓到底，切实兑现各项承诺，用实实在在的工作成效赢得群众的信任和支持。

我们要不断强化廉政建设，做到守纪律、讲规矩。全面落实党风廉政建设责任制，时刻把纪律和规矩挺在前面。严格执行“三重一大”制度，强化政府权力运行监督，以权力瘦身为廉政强身。坚持源头预防，加强公职人员廉洁从政教育。强化审计监督和行政监察，坚决惩治腐败。厉行节约，反对浪费，严控论坛节庆活动，严控政府投资项目规模，严控政府一般性支出。上半年完成公务用车改革任务。扎紧制度笼子，坚决打掉权力寻租空间，严肃查处发生在群众身边的腐败问题，努力做到干部清正、政府清廉、政治清明。

各位代表，新的起点开启新的征程。时代的要求，人民的期望，激励我们不懈奋进。当前，鄂尔多斯正迈步走在全面建成小康社会的康庄大道上，任何艰难险阻都阻挡不了我们前进的步伐。让我们在市委的坚强领导下，以更加坚定的意志攻坚克难，以更加饱满的热情开拓创新，以更加务实的作风勤勉工作，奋力开创全市经济社会发展新局面，为把鄂尔多斯建成祖国北疆亮丽风景线上璀璨明珠而努力奋斗！
